# Evaluating the Impact of Hyperthermic Intraperitoneal Chemotherapy (HIPEC) on Interval and Secondary Debulking in Ovarian Cancer: A Systematic Review

**DOI:** 10.3390/cancers17050904

**Published:** 2025-03-06

**Authors:** Dimitrios Tsolakidis, Dimitrios Kyziridis, Theodoros Panoskaltsis, Apostolos Kalakonas, Vasileios Theodoulidis, Kimon Chatzistamatiou, Dimitrios Zouzoulas, Antonios-Apostolos Tentes

**Affiliations:** 1 1st Department of Obstetrics & Gynecology, Aristotle University of Thessaloniki, 56429 Thessaloniki, Greece; theodoulidisvasilis@yahoo.gr (V.T.); kimon.chatzistamatiou@gmail.com (K.C.); dzouzoulas@hotmail.gr (D.Z.); 2 Surgical Department of Peritoneal Surface Malignancy Program, EUROMEDICA Kyanous Stavros, 54636 Thessaloniki, Greece; dkyziridis@gmail.com (D.K.); panoskaltsistheo@gmail.com (T.P.); apostoliskal@gmail.com (A.K.); tolistentes@gmail.com (A.-A.T.)

**Keywords:** hyperthermic intraperitoneal chemotherapy (HIPEC), ovarian cancer, cytoreductive surgery (CRS), interval debulking, secondary debulking, progression-free survival (PFS), overall survival (OS), surgical oncology, oncologic outcomes

## Abstract

**Background/Objectives:** Hyperthermic intraperitoneal chemotherapy (HIPEC) was revealed as a promising adjunct to cytoreductive surgery (CRS) in the treatment of advanced epithelial ovarian cancer (EOC). This review evaluated the impact HIPEC had on survival outcomes, recurrence patterns and safety in patients that underwent HIPEC in conjunction with interval and secondary CRS for advanced and recurrent ovarian cancer. **Methods:** A thorough search was conducted using PubMed, Scopus, Cochrane Library, and Google Scholar to identify relevant studies published until 1 January 2025. The studies were assessed for survival outcomes, recurrence patterns, safety, and quality of life. The risk of bias was evaluated using the ROB 2 tool for randomized and ROBINS-I for non-randomized articles. The results are presented narratively, highlighting key findings, comparing results and assessing inconsistencies and limitations. **Results:** HIPEC demonstrated significant improvements in progression-free survival (PFS) and overall survival (OS), particularly in cases with optimal cytoreduction (CC-0/CC-1). The recurrence patterns showed a reduction in peritoneal dissemination with HIPEC, although extraperitoneal recurrences were reported in some cases. Most studies reported comparable morbidity rates between HIPEC and non-HIPEC groups, with acceptable safety profiles. The variability in the HIPEC protocols and the limited quality-of-life and cost-effectiveness data were noteworthy limitations. **Conclusions:** HIPEC, when performed during interval or secondary CRS, offers survival benefits and can modify recurrence patterns in advanced EOC, although challenges related to protocol standardization, patient selection, and long-term outcomes persist. Future research should focus on multicenter trials with uniform protocols, long follow-up periods and patient-centered outcomes to further validate the role of HIPEC in clinical practice.

## Simple Summary

Advanced ovarian cancer remains one of the most challenging malignancies to treat, with high recurrence rates despite modern therapeutic choices. Hyperthermic intraperitoneal chemotherapy (HIPEC) is a novel treatment approach that is delivered directly into the abdominal cavity during surgery to target residual cancer cells. This narrative review evaluated the effectiveness of HIPEC combined with surgery and investigated its potential to improve survival, reduce recurrence, and improve its overall safety. By analyzing the published results of 16 studies, we looked into the benefits, challenges, and future directions of HIPEC in managing advanced ovarian cancer.

## 1. Introduction

### 1.1. Rationale

Despite the significant advances in the treatment of epithelial ovarian cancer (EOC), a considerable percentage of patients present with advanced-stage disease or with recurrence after initial therapy [1]. As the leading cause of gynecological cancer mortality, EOC continues to challenge the results of oncologic interventions. Even though primary cytoreductive surgery (CRS) followed by systemic chemotherapy has remained the cornerstone of management, the prognosis for patients with residual or recurrent disease remains suboptimal [2]. Novel therapeutic approaches are critical to address the limitations of current treatment strategies.

Intraperitoneal chemotherapy has gained attention due to its capacity to deliver high drug concentrations directly to the peritoneal cavity while minimizing systemic toxicity [3]. However, its widespread adoption has been limited due to issues of tolerability and treatment-related complications [4].

Hyperthermic intraperitoneal chemotherapy (HIPEC) was proposed as a promising adjunct to CRS in the management of EOC [5]. This technique involves the direct perfusion of heated chemotherapeutic agents into the peritoneal cavity after the completion of cytoreductive surgery CC-0 or CC-1 (using the completeness of cytoreduction (CC) score), where hyperthermia is used to enhance the cytotoxicity and facilitate deeper penetration of the chemotherapeutic agents into residual tumor tissue less than 2.5 mm, overcoming the limitations of traditional intraperitoneal therapies [6]. The peritoneum is a membrane of a wide surface and is selected by very resistant clones, limiting the results of intravenous chemotherapeutic agents [7]. By targeting microscopic peritoneal disease, HIPEC offers a theoretical advantage in reducing the risk of recurrence and preserving systemic tolerability [8].

The direct delivery of chemotherapeutics into the peritoneal cavity, results in significantly higher local drug concentrations compared with systemic administration, while the application of heat further enhances the drug penetration into peritoneal surfaces [9]. Hyperthermia also disrupts cellular homeostasis by impairing DNA repair mechanisms, such as homologous recombination, making cancer cells susceptible to chemotherapy-induced damage [10]. Elevated temperatures increase the membrane permeability, facilitating deeper drug absorption into tumor tissues [11]. Beyond direct cytotoxicity, hyperthermia triggers the activation of heat shock proteins, which contribute to immune modulation and may enhance systemic anti-tumor responses [12].

### 1.2. Objectives

This review aimed to investigate the safety, efficacy and clinical outcomes associated with HIPEC combined with both interval and secondary CRS, based on data from the published literature. By analyzing the available studies, this review sought to clarify the potential role of this promising combined approach in extending survival and improving the quality of life for patients with advanced-stage ovarian cancer.

## 2. Materials and Methods

### 2.1. Eligibility Criteria

The study types eligible for inclusion were randomized clinical trials, cohort studies and case-control studies. the studies need to have specifically investigated the use of HIPEC combined with CRS in patients diagnosed with primary or recurrent epithelial ovarian cancer that underwent interval or secondary debulking to be included in this review. The articles had to report sufficient outcome data, including the overall survival (OS), progression-free survival (PFS), perioperative morbidity, or treatment-related complications. Studies that involved fewer than ten patients, abstract-only presentations, and editorial letters or articles that did not differentiate EOC data from other malignancies were excluded. For studies performed at the same institution with overlapping data, only the most recent or methodologically robust one was included to avoid duplication.

### 2.2. Information Sources

In order to evaluate the role of HIPEC in combination with cytoreductive surgery for epithelial ovarian cancer, a thorough literature search was conducted using established databases. These were PubMed, Cochrane Library, Scopus, and ClinicalTrials.gov. The search strategy consisted of the following key terms: “Hyperthermic Intraperitoneal Chemotherapy”, “HIPEC”, “Cytoreductive Surgery”, “Debulking”, and “Ovarian Cancer”, utilizing both free-text and MeSH terms to maximize the coverage. The search targeted studies written in English language that were published until 1 January 2025. Additional studies were identified by manually screening the references of selected articles to ensure all eligible studies were included.

### 2.3. Search Strategy

The exact search strategy is presented below.

Pubmed (644 studies): (“Hyperthermic Intraperitoneal Chemotherapy” OR “HIPEC” OR “Hyperthermic Intraperitoneal Chemotherapy”[MeSH Terms]) AND (“Cytoreductive Surgery” OR “Debulking” OR “Cytoreduction Surgical Procedures”[MeSH Terms]) AND (“Ovarian Cancer” OR “Ovarian Neoplasms”[MeSH Terms]).

Cochrane Library (165 studies): ((“Hyperthermic Intraperitoneal Chemotherapy” OR “HIPEC”) AND (“Cytoreductive Surgery” OR “Debulking”) AND “Ovarian Cancer”):ti,ab,kw.

Scopus (794 studies): TITLE-ABS-KEY ((“Hyperthermic Intraperitoneal Chemotherapy” OR “HIPEC”) AND (“Cytoreductive Surgery” OR “Debulking”) AND “Ovarian Cancer”).

ClinicalTrials.gov (68 studies): (“Hyperthermic Intraperitoneal Chemotherapy” OR “HIPEC”) AND (“Cytoreductive Surgery” OR “Debulking”) AND “Ovarian Cancer”.

The filters that were applied were for English language and the human female population.

### 2.4. Selection Process

Two independent reviewers performed an initial screening based on the titles of the studies and their abstracts to identify potentially relevant studies. Afterward, full-text articles of these studies were reviewed independently by the two reviewers to assess their eligibility. Any disagreements between the two reviewers in the selection process were resolved by consensus with a third investigator. 

### 2.5. Data CollectionProcess

A standardized data extraction form was employed to ensure uniformity in capturing the critical study characteristics. The data extraction was performed by two independent reviewers and the data were confirmed by comparing the forms. If there were any differences in values, a third reviewer evaluated the values. 

### 2.6. Data Items

The data extracted from the enrolled studies included the following:

- Publication details (author, year);
- Study design (randomized controlled trials, cohort studies, case-control studies);
- The size of the patient population (sample size);
- The time of people enrollment (recruitment period);
- Details of the intervention (HIPEC drug, duration, temperature, chemotherapy agent, follow-up);
- Clinical outcomes (PFS, OS, recurrence patterns).

### 2.7. Study Risk of Bias Assessment

The risk of bias in the included studies was assessed by two independent reviewers using the Risk of Bias 2.0 (RoB 2.0) tool for the randomized controlled trials and the Risk of Bias in Non-Randomized Studies of Interventions (ROBINS-I) tool for the non-randomized studies. For the randomized studies, five domains were evaluated: bias regarding the randomization process, deviations from intended interventions, missing outcome data, measurements of the outcome, and selection of the reported result. For non-randomized studies, we assessed the following seven domains: bias due to confounding, classification of interventions, selection of participants, deviations from intended interventions, missing data, measurement of outcomes, and selection of the reported results. Disagreements in the bias judgments were resolved by consensus. These tools ensured a thorough evaluation of the methodological quality and integrity of the studies.

### 2.8. Effect Measures

The outcomes of the effect measures were reported for the PFS in months; for the OS in months; and for the recurrence patterns, either months, percentage of patients, or narratively, depending on the report of each study.

### 2.9. Synthesis of Results

The results were synthesized narratively, grouping studies by the type of CRS (interval or secondary debulking) and their reported outcomes (survival, recurrence, safety). Due to significant heterogeneity in the study designs, patient populations, and HIPEC protocols that were used across the studies, a meta-analysis was considered not feasible.

### 2.10. Certainty Assessment

In order to assess the certainty of the evidence, our team used the GradeGDTpro tool to separately evaluate each of the outcomes across studies and based on the study types. Two independent reviewers made their report after carefully giving a grade in each of the domains of certainty, and any disagreements were resolved by a consensus from a third reviewer. The five domains investigated by this assessment were the risk of bias, inconsistency, indirectness, imprecision and other considerations, which resulted in the evaluation of the certainty of each piece of evidence, graded as “very low”, “low”, “moderate”, and “high”.

## 3. Results

### 3.1. Study Selection

**Figure 1:**
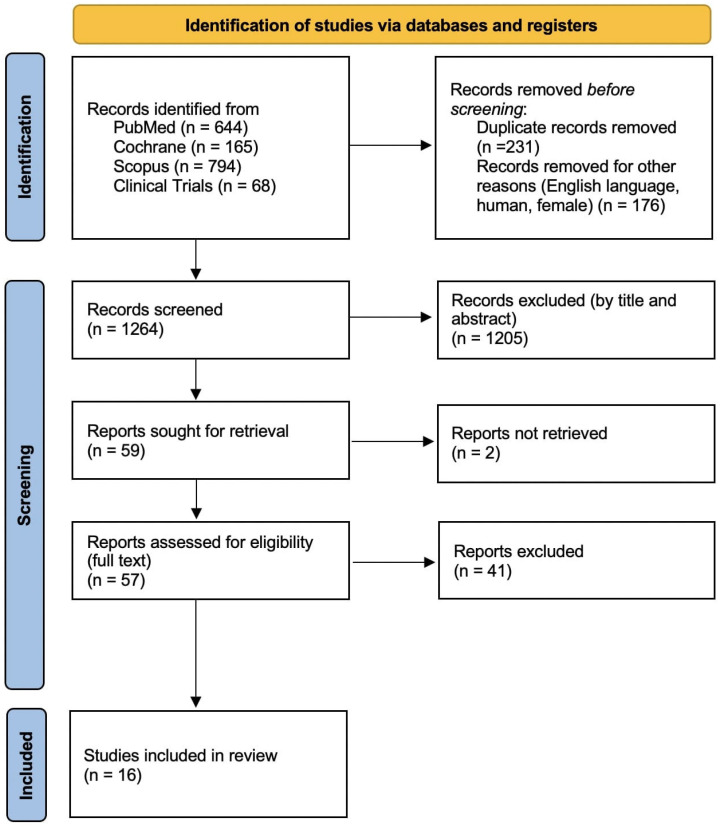
PRISMA flow chart.

The studies that the search strategy identified were evaluated and screened independently by two reviewers. The initial search resulted in 1671 articles. In detail, the Pubmed database search resulted in 644 articles, the Cochrane database search in 165 studies, the Scopus database search in 794 studies, and the ClinicalTrials.gov search presented another 68 clinical trials. After removing the duplicate studies and filtering for English language and human female subjects, 1482 articles remained. The study screening based on the title and abstract content resulted in 59 studies. Two studies could not be retrieved in full text. Finally, after reviewing the final articles based on their full text, the total number of studies that remained for inclusion in this review were 16. The flow chart of the search strategy is presented below (Figure 1). 

### 3.2. Study Characteristics

The 16 studies included in this review, namely (presented in chronological order based on the time of publication) Spiliotis 2015 [13], Ceresoli 2018 [14], Van Driel 2018 [15], Jou 2021 [16], Chambers 2021 [17], Marielli 2021 [18], Zivanovic 2021 [19], Kim 2022 [20], Antonio 2022 [21], Kim-Chun 2023 [22], Lee 2023 [23], Classe 2024 [24], Campos 2024 [25], Ghirardi 2024 [26], Wang 2024 [27] and Fagotti 2024 [28], were studies that investigated and have reported the results of the addition of HIPEC to the interval or secondary CRS for ovarian cancer. These articles describe a variety of study designs, including randomized controlled trials (RCTs), prospective and retrospective cohort studies and retrospective case–control studies. 

The following tables summarize the studies included in this review and their main characteristics, arranged based on the type of CRS used in each study (interval, secondary, both interval and secondary) (Table 1, Table 2 and Table 3). 

### 3.3. Risk of Bias Assessment

We evaluated the risk of bias in the included studies using two different tools based on the design of each study; the RoB 2.0 tool was used for the randomized controlled trials and the ROBINS-I tool for the non-randomized studies.

Regarding the randomized trials, the RoB 2.0 tool targets five bias domains: bias arising from the randomization process, bias from deviations of intended interventions, bias due to missing outcome data, bias regarding the measurement of the outcomes, and bias regarding the selection of the reported result.

For the non-randomized studies, we used the ROBINS-I tool, which examines seven domains of possible bias: risk of bias due to confounding; risk of bias in the classification of interventions; risk of bias in the participant selection; risk of bias due to deviations from intended interventions; risk of bias due to missing data; risk of bias arising from the measurement of outcomes and; finally, bias regarding the selection of the reported result. 

**Figure 2:**
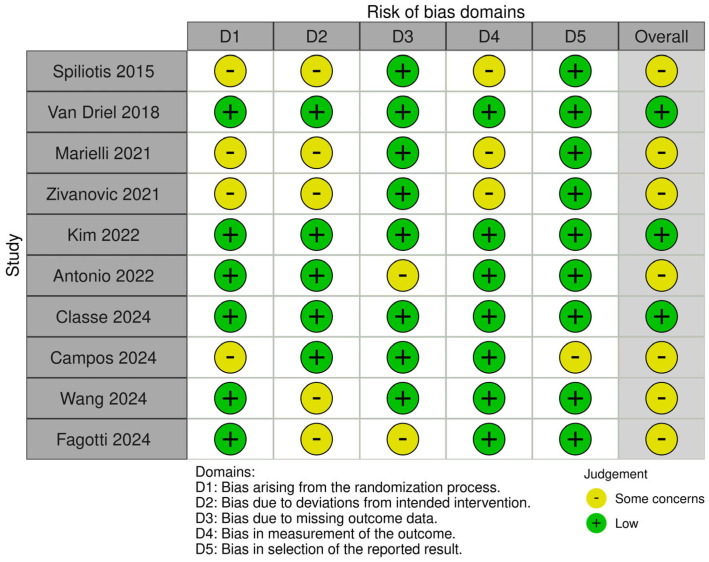
Traffic light plot of randomized studies [13,15,18,19,20,21,24,25,27,28].

**Figure 3:**
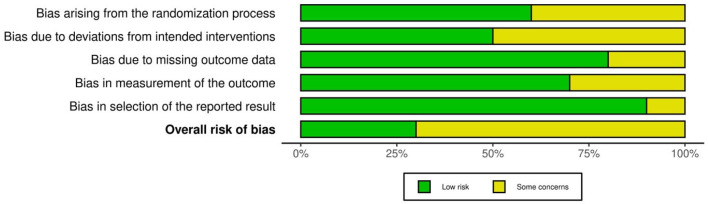
Summary plot of randomized studies.

**Figure 4:**
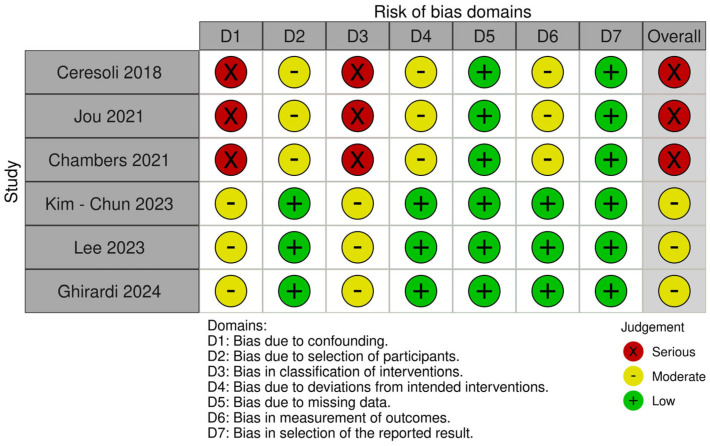
Traffic light plot of non-randomized studies [14,16,17,22,23,26].

**Figure 5:**
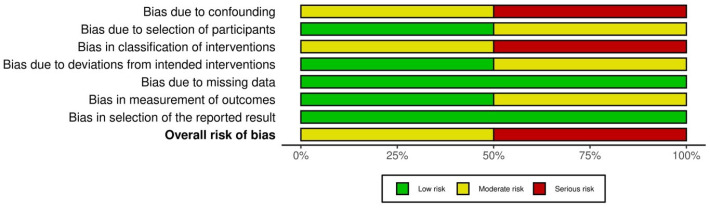
Summary plot of non-randomized studies.

By applying these tools we aimed to provide a consistent evaluation of the methodology and reliability of the included studies. The results of the assessments are summarized as traffic light plots and summary plots in the following figures (Figure 2, Figure 3, Figure 4 and Figure 5). 

### 3.4. Results of Individual Studies

#### 3.4.1. Study Designs and Populations

The 16 articles included in this review consisted of a variety of study designs, from randomized controlled trials to prospective and retrospective cohorts and case–control studies. This broad spectrum of methodologies provided a wide evaluation of HIPEC in both interval and secondary debulking for ovarian cancer under different settings.

Seven studies had randomized controlled designs. These studies, specifically Spiliotis 2015 [13], Van Driel 2018 [15], Zivanovic 2021 [19], Kim 2022 [20], Antonio 2022 [21], Classe 2024 [24], and Fagotti 2024 [28] (HORSE MITO-18 study), explored the efficacy of HIPEC in either interval or secondary debulking surgery and reported outcomes including progression-free survival and overall survival. These studies enrolled patients with advanced-stage and/or platinum-sensitive recurrent ovarian cancer, typically following strict inclusion criteria such as the absence of extra-abdominal metastases or suitability for optimal cytoreductive surgery. While these trials offer high-level evidence, their designs varied in terms of HIPEC protocols used, patient stratification and the incorporation of modern systemic therapies.

Four studies were prospective cohort analyses (Kim-Chun 2023 [22], Lee 2023 [23], Ghirardi 2024 [26], and Campos 2024 [24]). These studies primarily evaluated the outcomes of HIPEC in selected patients that underwent interval or secondary cytoreduction, and investigated the feasibility, safety, and survival impact of HIPEC, which complemented the evidence from randomized trials.

The remaining five studies were of retrospective designs. Ceresoli 2018 [14] and Jou 2021 [16] utilized case–control and retrospective cohort designs, respectively, to assess the long-term benefits of HIPEC in secondary debulking, particularly focusing on recurrence patterns and survival outcomes. Chambers 2021 [17] and Marielli 2021 [18] were retrospective studies that reported on outcomes of HIPEC in advanced or recurrent ovarian cancer, with a focus on feasibility and safety. Finally, Wang 2024 [27] provided a retrospective evaluation of HIPEC in conjunction with interval cytoreduction, with a particular focus on pathological and clinical responses, rather than long-term survival metrics.

Across these studies, the populations included predominantly patients with high-grade serous ovarian cancer (HGSOC) in advanced stages (FIGO III–IV) who were candidates for optimal cytoreduction (CC-0 or CC-1). Several studies, including Zivanovic 2021 [19] and Fagotti 2024 [28], specifically targeted patients with platinum-sensitive recurrent disease, while Ghirardi 2024 [26] extended the inclusion to more challenging cases such as those with extended cycles of neoadjuvant chemotherapy or FIGO stage IV disease. It should be noted that FIGO staging for ovarian cancer changed in 2014, even though some studies enrolled patients as early as 2007, so the staging methods across some studies were not under the same guidelines. This heterogeneity highlights how different patient selection and treatment approaches might be reflecting disease and treatment complexities and the evolving role of HIPEC in ovarian cancer management.

#### 3.4.2. HIPEC Protocols and Chemotherapeutic Agents

The HIPEC protocols and chemotherapeutic regimens used across the 16 studies were of great variability in terms of the agent selection, dosage, temperature, duration, and delivery method.

Cisplatin was the most frequently used chemotherapeutic agent, featured in 10 out of the 16 studies, namely, Spiliotis 2015 [13], Van Driel 2018 [15], Chambers 2021 [17], Marielli 2021 18], Kim 2022 [20], Kim-Chun 2023 [22], Antonio 2022 [21], Classe 2024 [24], Fagotti 2024 [28] and Ghirardi 2024 [26]. The dosages ranged between 75 mg/m^2^ (e.g., Antonio 2022 [21], Classe 2024 [24], Fagotti 2024 [28]) and 100 mg/m^2^ (Van Driel 2018 [15]), and usually administered for durations of 60 to 90 min at intraperitoneal temperatures of 41–43 °C. These studies validated cisplatin’s cytotoxic efficacy and established itself as a standard HIPEC agent.

Carboplatin was exclusively used by Zivanovic 2021 [19], being administered at a dose of 800 mg/m^2^ for 90 min at a temperature of 41–43 °C. The choice of carboplatin in this study was likely due to its lower nephrotoxicity compared with cisplatin, making it suitable for specific patient populations, particularly those with pre-existing renal concerns.

Paclitaxel was the primary agent in two studies (Wang 2024 [27], Campos 2024 [25]) that explored its role as an alternative to platinum-based agents, with a focus on its microtubule-stabilizing properties. In Wang 2024 [27], paclitaxel was delivered at a dose of 175 mg/m^2^ over 90 min at a temperature of 42 °C. Similarly, Campos 2024 [25] utilized paclitaxel (175 mg/m^2^) for 60 min at a slightly higher temperature of 42–43 °C.

Regarding the delivery methods, most studies, including Van Driel 2018 [15], Zivanovic 2021 [19], Kim 2022 [20], Classe 2024 [24], Campos 2024 [25], and Fagotti 2024 [28], used a closed-abdomen technique. This method minimizes spillage, reduces exposure risks, and allows the heat to be distributed evenly. In contrast, Spiliotis 2015 [13], Antonio 2022 [21], and Marielli 2021 [18] used the open “Coliseum” technique, which allowed for direct visualization and manipulation of the peritoneal cavity during HIPEC. These methodological differences were often driven by institutional expertise and surgeon preferences.

The temperature and duration parameters were relatively consistent across the studies. The intraperitoneal temperatures were maintained in a range of 41–43 °C, which maximized the cytotoxicity and avoided thermal injury to the surrounding tissue. The duration of HIPEC varied from 60 min, as seen in Antonio 2022 [21], Classe 2024 [24], and Fagotti 2024 [28], to 90 min in studies such as Van Driel 2018 [15], Zivanovic 2021 [19], Kim 2022 [20], Wang 2024 [27], and Kim-Chun 2023 [22].

Postoperative systemic chemotherapy regimens were reported in multiple studies. Carboplatin and paclitaxel combinations were the most common regimens used, as seen in Ghirardi 2024 [26], Marielli 2021 [18], and Kim 2022 [20]. Some studies, such as Chambers 2021 [17] and Kim-Chun 2023 [22], provided insights in HIPEC’s feasibility without uniform systemic therapy reporting. In addition, studies like Fagotti 2024 [28] and Antonio 2022 [21] highlighted the importance of including HIPEC into treatment strategies, emphasizing its role as an adjunct rather than a standalone intervention.

All included studies emphasized that HIPEC’s efficacy relates highly to achieving complete or near-complete cytoreduction (CC-0 or CC-1). Even though the diversity found across the studies concerning HIPEC protocols severely damaged the generalizability of the results, at the same time, it points to the need for further standardization to optimize outcomes and ensure consistency across clinical settings.

#### 3.4.3. Results on Interval and Secondary Debulking with HIPEC

The results reported from the studies indicate the role of HIPEC in improving the outcomes for patients undergoing interval and secondary cytoreductive surgery in advanced-stage ovarian cancer. 

The efficacy of HIPEC in interval CRS was demonstrated in several studies. Van Driel 2018 [15], a pivotal RCT, showed a significant improvement in the median PFS in the HIPEC group (14.2 months) compared with the control group (10.7 months, HR 0.66, *p* = 0.003). The overall survival was also significantly longer in the HIPEC group, with a median OS of 45.7 months compared with 33.9 months in the control arm (HR 0.67, *p* = 0.02). A recent follow-up analysis published by Aronson et al., 2023 [2] extended these findings, reporting 10-year OS outcomes. The HIPEC group demonstrated a 10-year OS rate of 24.6% compared with 13.1% in the control group, which strengthened the conclusions of the initial study and confirmed the survival benefit of HIPEC over the long term [29].

The Kim 2022 [20] study similarly used an RCT design and focused on the health-related quality of life (HRQoL) and the feasibility of HIPEC. Although it did not provide specific PFS or OS data values, the study demonstrated the safety and tolerability of the technique with no significant differences in the HRQoL between the HIPEC and control groups.

Lee 2023 [23], a large prospective cohort study, demonstrated significant survival advantages in the HIPEC arm. They used either cisplatin (75 patients) or paclitaxel (34 patients), and found no significant difference in the PFS and OS between the two drugs. The median PFS in the HIPEC group was 22.9 months versus 14.2 months in the non-HIPEC group (HR 0.61, *p* = 0.005), while the median OS was not reached in the HIPEC group compared to with 53.0 months in the control group (HR 0.31, *p* = 0.002). Recurrence patterns were also affected, with significantly fewer peritoneal recurrences in the HIPEC arm (32.8% vs. 64.1%, *p* = 0.001), although the extraperitoneal recurrences were slightly more frequent.

The RCT of Antonio et al. in 2022 [21] reported a statistically significant improvement in the PFS with HIPEC (18 months vs. 12 months, *p* = 0.038) and a trend toward improved OS (52 months vs. 45 months), although this difference was not statistically significant. Additionally, the patients with supramesocolic disease showed an extended PFS of 24.1 months versus 9.4 months in the control group (*p* = 0.031).

The phase II trial of Marielli et al. in 2021 [18] provided feasibility data rather than detailed survival outcomes, reporting successful HIPEC administration in all patients and a 5-year OS rate of 42 ± 8%.

Kim-Chun 2023 [22] focused on cost-effectiveness and survival, reporting a significant reduction in the PFS and OS hazards with HIPEC (HR for PFS: 0.60, *p* = 0.04; HR for OS: 0.53, *p* = 0.04).

Wang 2024 [27] performed a trial where the patients in the HIPEC group received HIPEC during initial laparoscopic exploration; afterwards they received three cycles of intravenous chemotherapy, followed by interval debulking and HIPEC. Although it was an interim analysis, it highlighted superior pathological complete response rates in the HIPEC arm using Chemotherapy Response Scores (CRS-1, CRS-2, CRS-3) to evaluate the response to chemotherapy, with CRS-3 proposed to serve as an alternative indicator for PFS. This study reported CRS-3 for the HIPEC arm in 20.5% vs. 4.8% for the non-HIPEC arm (*p* < 0.05), suggesting beneficial long-term outcomes.

HIPEC during secondary CRS was evaluated in several studies, with mixed results. Spiliotis 2015 [13] demonstrated a significant OS benefit with the HIPEC group, where they achieved a mean OS of 26.7 months compared with 13.4 months for the control group (*p* = 0.006). Among the platinum-sensitive patients, this benefit was particularly pronounced (26.8 months vs. 15.2 months, *p* = 0.035).

However, the Zivanovic 2021 [19] study found no significant improvement in the PFS (12.3 months in the HIPEC group vs. 15.7 months in the controls, HR 1.54, *p* = 0.05) or OS (52.5 months vs. 59.7 months, HR 1.39, *p* = 0.31), highlighting the variability in the outcomes.

The CHIPOR trial (Classe 2024 [24]) demonstrated modest gains in the PFS (10.2 months vs. 9.5 months, HR 0.79, *p* < 0.05) but significant OS improvements in the HIPEC group (54.3 months vs. 45.8 months, HR 0.73, *p* = 0.024). Similarly, Fagotti 2024 (HORSE MITO-18 trial) reported improved post-recurrence survival but no significant difference in the PFS (25 months in the HIPEC group vs. 23 months in the controls).

Retrospective studies, such as Ceresoli 2018 [14], supported the survival benefits of HIPEC, with a median OS not reached in the HIPEC group compared with 32.5 months in the controls (*p* = 0.048). Jou et al. in 2021 [16], however, found no significant differences in the PFS or OS, with a concerning increase in the platinum-resistant recurrence rates in the HIPEC group (50% vs. 23%, *p* = 0.024). Chambers 2021 [17] highlighted altered recurrence patterns, with increased extraperitoneal recurrences following HIPEC.

Studies that evaluated both interval and secondary CRS provided additional insights. Ghirardi 2024 [26] reported a median PFS of 24.0 months but did not find significant differences based on the FIGO stage or HIPEC application. Campos 2024 [25] demonstrated a median PFS of 23 months in the HIPEC group compared to with 19 months in the controls, alongside modest OS improvements (48 months vs. 46 months).

The safety profile of HIPEC was consistent across the studies. Severe postoperative complications (Clavien–Dindo grade ≥ 3) were observed in 10–28% of patients, with no significant differences compared with the control groups. Morbidity was most commonly hematological or related to wound healing. the quality of life was generally unaffected by HIPEC, as shown in studies such as Kim 2022 [20] and Classe 2024 [24].

The main results are seen in the following table (Table 4).

### 3.5. Certainty of Evidence

The certainty of evidence provided was rated as very low in each of the reported outcomes. In all the domains of risk of bias, inconsistency, indirectness, imprecision, and other considerations (regarding mainly the publication bias), the grouped evidence of the studies was graded very low regarding its certainty of evidence, highlighting the vast diversity between the studies and the inconsistency and low quality of most of the designed studies. The summary of findings table is presented below (Table 5).

### 3.6. Overall

This review highlighted the significant role of HIPEC in improving outcomes for advanced ovarian cancer patients that underwent interval and secondary cytoreductive surgeries. Across the 16 studies analyzed, randomized trials such as Van Driel 2018 [15], Classe 2024 [24], and Spiliotis 2015 [13], consistently demonstrated survival benefits, with notable improvements in progression-free and overall survival, while observational studies, including Ghirardi 2024 [26] and Chambers 2021 [17], further supported these findings, where they provided actual evidence of HIPEC’s feasibility and safety. While variability in the HIPEC protocols and patient populations exists, the cumulative evidence underscores its potential to benefit survival and recurrence patterns when integrated into EOC treatment strategies.

## 4. Discussion

The findings of this narrative review underline the evolving role of hyperthermic intraperitoneal chemotherapy in combination with interval or recurrent cytoreductive surgery as a treatment strategy for epithelial ovarian cancer. Despite significant advancements in the management of EOC, including optimal CRS followed by platinum-based chemotherapy, the prognosis of advanced-stage disease remains poor. Studies indicate that approximately 70% to 80% of patients experience disease recurrence within five years following initial treatment [30]. This dictates the investigation of new therapeutic tools, such as HIPEC, that can potentially improve survival outcomes while preserving quality of life.

### 4.1. Survival Outcomes: A Consistent Theme with Variability

The survival benefits of HIPEC were prominent across most of the studies reviewed. Randomized controlled trials such as Van Driel 2018 [15] and its 10-year follow-up reported by Aronson 2023 [2] provided strong evidence of improved PFS and OS in patients that underwent interval CRS with HIPEC. Similar findings were reported in the studies of Classe 2024 [24] and Spiliotis 2015 [13], which extended these benefits to secondary debulking settings. Observational studies, including Ceresoli 2018 [14] and Chambers 2021 [17], agreed with these results, emphasizing altered recurrence patterns with reduced peritoneal disease.

However, the results of this review, as addressed previously, also underscores the variability. Studies such as Zivanovic 2021 [19] and Jou 2021 [16] reported no significant improvement in the PFS or OS, highlighting the heterogeneity in patient populations, such as differences in the platinum sensitivity and residual disease status, which likely influenced the outcomes. These discrepancies probably point to the critical importance of patient selection. The inclusion of heterogeneous populations in some studies, spanning FIGO stages III and IV, as well as platinum-resistant and platinum-sensitive diseases, complicated the interpretation of the results and emphasized the need for uniformity in the methodology.

### 4.2. Impact on Recurrence Patterns

HIPEC’s effect on disease recurrence patterns further strengthens its potential as an addition to CRS. Several studies, including Van Driel 2018 [15] and Lee 2023 [23], reported significantly lower rates of peritoneal recurrence in HIPEC-treated patients, which aligns with the hypothesized mechanisms of direct cytotoxicity to residual microscopic disease and the prevention of tumor reimplantation. However, the increased rates of extraperitoneal recurrence observed in studies such as Chambers 2021 [17] and Ghirardi 2024 [26] are definitely alarming. While HIPEC may effectively control locoregional disease, its effects on the systemic disease spread remain limited, implying the need for concurrent systemic therapies.

### 4.3. Safety and Feasibility: A Balanced Perspective

The safety of HIPEC is a critical factor in its wider adoption. Across the analysed studies of this review, HIPEC demonstrated an acceptable safety profile, with rates of grade 3–4 complications comparable with standard CRS. Van Driel 2018 [15], Classe 2024 [24], and Antonio 2022 [21] consistently reported no significant increases in severe adverse events, although they reported extended operative times and hospital stays. Notably, Kim 2022 [20] demonstrated that HIPEC does not negatively impact the health-related quality of life (HRQoL), a very important consideration for patients with advanced cancer.

Nevertheless, the variability in the morbidity rates across the studies dictates the need for further investigation. For instance, Wang 2024 [27] and Marielli 2021 [18] highlighted the potential for renal toxicity with cisplatin-based HIPEC, especially for patients with preexisting comorbidities. Additionally, the technical complexity of HIPEC necessitates a highly skilled surgical team and specialized infrastructure, which may limit its feasibility in less funded settings.

### 4.4. Variability in Protocols and Patient Selection

A key challenge identified in this review was the significant variability in HIPEC protocols. While cisplatin was the most commonly used agent, the dosages, durations, and temperatures used varied widely, where they ranged from 60 to 90 min and 41 °C to 43 °C. Alternatives such as paclitaxel, which was used in the Wang 2024 [27] and Campos 2024 [25] studies, suggest that different agents may offer comparable efficacy. However, this lack of standardization complicated the synthesis of evidence, limited the feasibility of direct comparisons and hindered the identification of optimal HIPEC regimens.

Patient selection also emerged as a pivotal factor. Most studies, including Kim-Chun 2023 [22] and Lee 2023 [23], included only patients who achieved optimal cytoreduction (CC-0/CC-1), which limited the generalizability of findings to patients with residual disease. Furthermore, studies like Spiliotis 2015 [13], which included both platinum-sensitive and platinum-resistant populations, introduced additional heterogeneity. Refined criteria have to be formulated to identify the patients most likely to benefit from HIPEC.

### 4.5. Role of Long-Term Follow-Up

While studies like Aronson 2023 [2] provided critical insights into the long-term survival benefits of HIPEC, most trials reported outcomes over a median of 2–5 years follow-up, which may not fully capture its impact on late recurrences and survival. The lack of long-term follow-up data in crucial studies, such as Wang 2024 [27] and Fagotti 2024 [28] limited the ability to evaluate HIPEC’s durability, particularly in secondary debulking settings where recurrence patterns may differ. Future trials with extended follow-up seem essential to address these gaps.

### 4.6. Integration with Modern Systemic Therapies

The availability of modern targeted therapies and maintenance strategies, such as poly-ADP-ribose-polymerase (PARP) inhibitors and bevacizumab, raises important questions about the integration of HIPEC into contemporary treatment strategies. While none of the reviewed studies explicitly addressed these combinations, the potential for synergistic effects seems worth exploring. Stratifying patients based on biomarkers such as BRCA mutation status and homologous recombination deficiency (HRD) could further optimize the application of HIPEC and improve outcomes.

### 4.7. Economic Considerations

The economic implications of HIPEC remain an underexplored area, where only a few studies, such as Kim-Chun 2023 [22], addressed its cost-effectiveness. The high costs associated with HIPEC, including prolonged operative times, specialized equipment, and intensive perioperative care, may pose significant limits to its use, particularly in resource-limited settings. Comprehensive cost-effectiveness analyses would be useful to evaluate its feasibility across diverse healthcare systems.

### 4.8. Limitations of Included Evidence

The studies included in this review offered helpful insights to understand the role of HIPEC in interval and secondary debulking surgeries for ovarian cancer, yet they were not without limitations and challenges. Critical issues were the heterogeneity of the study designs, patient populations, HIPEC protocols, and reported outcomes, diversities that complicated direct comparisons and limited the generalizability of findings.

One critical limitation was the inconsistency in the HIPEC protocols. Although most studies utilized cisplatin-based chemotherapy, differences in the drug combinations, temperatures, and durations were found. For example, while Van Driel 2018 [15] and Lee 2023 [23] used a standardized 90 min protocol with cisplatin at 41–43 °C, Antonio 2022 [21] employed a 60 min protocol, and Wang 2024 [27] used paclitaxel instead of cisplatin. Such differences raise questions about the optimal HIPEC agent and its applicability across diverse clinical settings.

Another challenge was the variability in the study designs and methodologies used. Randomized controlled trials, such as Van Driel 2018 [15] and Classe 2024 [24], provided high-level evidence, but their inclusion criteria often limited the external validity. On the other hand, retrospective studies, like Ceresoli 2018 [14] and Jou 2021 [16], were inherently prone to selection bias and confounding. The lack of standardized reporting for outcomes, such as recurrence patterns and health-related quality of life further affected the comprehensive evaluation.

Patient selection bias was another concern. Most studies included only patients who achieved optimal cytoreduction (CC-0 or CC-1), excluding those with residual disease, a fact that could overestimate the benefits of HIPEC in real-world practice, where complete cytoreduction is not always achievable. Moreover, the inclusion of platinum-sensitive and platinum-resistant populations in some studies, such as the study of Spiliotis et al., [13] in 2015, affects the interpretation of the results, as these included subgroups respond differently to HIPEC.

The lack of long-term follow-up data is also a significant limitation. While studies like Aronson 2023 [2] extended the follow-up of Van Driel 2018 [15] to 10 years, most trials reported outcomes over a median of 2–5 years, which is considered insufficient in order to fully capture the impact of HIPEC on the overall survival and late recurrence incidences. This gap points to the need for extended follow-up in future studies.

### 4.9. Limitations of Review Process

Even though the current review was conducted using a structured systematic approach, some limitations should be pointed out. First, our search strategy did not include grey literature and unpublished data, which even though it set a high standard for the studies eligible for inclusion, it could have excluded relevant and important available evidence. Another limitation arose from searching for studies written in English, which may have introduced a language bias and limited the representation of non-English speaking institutes. Finally, even though this review followed the principles of a systematic analysis, a meta-analysis was not conducted due to the significant heterogeneity in the study methodologies and reported outcomes, and instead a narrative approach was employed to interpret the results. Even though this approach leds to a thorough discussion, it did not quantify the effects estimates and could impact the broader applicability of the results. 

### 4.10. Future Directions

To address the limitations highlighted in this review, future research should focus on several key areas. First, multicenter randomized controlled trials with standardized protocols are essential to validate the observed survival benefits and establish optimal HIPEC regimens. Second, long-term follow-up studies are critical to understanding both the durability of HIPEC’s benefits and its impact on late recurrences. Third, incorporating biomarkers into patient selection criteria could assist in the identification of the candidates most likely to benefit from HIPEC. Finally, cost-effectiveness analyses and studies exploring the integration of HIPEC with modern systemic therapies are needed to guide its implementation in clinical practice.

## 5. Conclusions

This review demonstrated the potential benefits of HIPEC in conjunction with cytoreductive surgery as an approach in the management of epithelial ovarian cancer, with significant survival improvements in both OS and PFS, particularly in patients undergoing interval and secondary cytoreductive procedures. These benefits are greater in patients achieving complete cytoreduction, emphasizing the importance of surgical expertise and meticulous patient selection.

Even though the current data provides encouraging insights, certain limitations remain evident. The randomized controlled trials conducted formed a substantial part of the evidence base, but the variability in their HIPEC protocols and the inclusion of diverse patient populations complicated the synthesis of the findings. Moreover, the lack of sufficient quality-of-life data and cost-effectiveness analyses limited the interpretation of HIPEC’s benefits beyond survival outcomes. Future research should focus on further defining the role of HIPEC in specific patient subgroups, optimizing chemotherapeutic agents and determining the most effective timing of administration. Multicenter studies under standardized protocols with focus on long-term follow-up are crucial in order to prove HIPEC’s clinical utility.

In conclusion, HIPEC, when combined with CRS, holds the potential to improve the treatment of epithelial ovarian cancer. As ongoing research continues to shape the evidence base, its adoption should be guided by rigorous scientific evaluation and tailored to individual patient’s needs to maximize its clinical impact and utility.

## Figures and Tables

**Table 1:** Articles reporting results of HIPEC in interval debulking.

Study	Study Type	Sample	Recruitment Period	HIPEC Drug	Duration (min)	Temperature (°C)	Chemotherapy Agent	Follow-Up
Van Driel 2018 [15]	Randomized controlled trial	245 (123 HIPEC, 122 control)	2007–2016	Cisplatin	90	41–42	IV Carboplatin/ Paclitaxel	4.7 years
Marielli 2021 [18]	Phase II study	42 (No control)	2015–2020	Cisplatin	90	41–42	IV Carboplatin/ Paclitaxel	18 months
Kim 2022 [20]	Randomized controlled trial	92 (46 HIPEC, 46 control)	2010–2016	Cisplatin	90	41–42	IV Carboplatin/ Paclitaxel	12 months
Antonio 2022 [21]	Randomized phase III trial	71 (35 HIPEC, 36 control)	2014–2020	Cisplatin	60	42–43	IV Carboplatin/ Paclitaxel	10 years
Kim-Chun 2023 [22]	Prospective cohort	77 (No control)	2017–2022	Cisplatin	90	41–42	IV Carboplatin/ Paclitaxel	N/A
Lee 2023 [23]	Prospective cohort	196 (109 HIPEC, 87 control)	2017–2022	Cisplatin or Paclitaxel	90	41–43	IV Carboplatin/ Paclitaxel	28.2 months
Wang 2024 [27]	Randomized controlled trial	65 (43 HIPEC, 22 control)	2020–2023	Paclitaxel	90	42	IV Carboplatin/ Paclitaxel	Interim

**Table 2:** Article reporting results of HIPEC in secondary debulking.

Study	Study Type	Sample	Recruitment Period	HIPEC Drug	Duration (min)	Temperature (°C)	Chemotherapy Agent	Follow-Up
Spiliotis 2015 [13]	Randomized phase III trial	120 (60 HIPEC, 60 control)	N/A	Cisplatin	90	41–42	IV Carboplatin/ Paclitaxel	N/A
Ceresoli 2018 [14]	Retrospective case-control	56 (28 HIPEC, 28 control)	2010–2016	Cisplatin	90	41–42	IV Carboplatin/ Paclitaxel	43 months
Jou 2021 [16]	Retrospective cohort	68 (20 HIPEC, 48 control)	2010–2019	Cisplatin	N/A	N/A	IV Carboplatin/ Paclitaxel	Median 19.1 months
Chambers 2021 [17]	Retrospective cohort	92 (No control)	2014–2020	Cisplatin	90	41–43	IV Carboplatin/ Paclitaxel	2.3 years
Zivanovic 2021 [19]	Randomized phase II trial	98 (49 HIPEC, 49 control)	2015–2020	Carboplatin	90	41–43	IV Carboplatin/ Paclitaxel	24 months
Classe 2024 [24]	Randomized phase III trial	415 (207 HIPEC, 208 control)	2010–2021	Cisplatin	60	41	IV Carboplatin/ Paclitaxel	N/A
Fagotti 2024 [28]	Randomized phase III trial	167 (83 HIPEC, 84 control)	2017–2021	Cisplatin	60	41.5	IV Carboplatin/ Paclitaxel	N/A

**Table 3:** Article reporting results of HIPEC in both interval and secondary debulking.

Study	Study Type	Sample	Recruitment Period	HIPEC Drug	Duration (min)	Temperature (°C)	Chemotherapy Agent	Follow-Up
Campos 2024 [24]	Randomized phase III trial	76 (32 HIPEC, 44 control)	2014–2019	Paclitaxel	60	42–43	IV Carboplatin/ Paclitaxel	Median 24 months
Ghirardi 2024 [26]	Prospective cohort	205 (No control)	2019–2022	Cisplatin	90	41–43	IV Carboplatin/ Paclitaxel	24 months

**Table 4:** Summary of results of studies.

Study	PFS	OS	Recurrence Patterns
Spiliotis 2015 [13]	N/A	26.7 (HIPEC) vs. 13.4 months (control, *p* = 0.006)	N/A
Ceresoli 2018 [14]	13.96 (HIPEC) vs. 13.23 months (control, *p* = 0.454)	Not reached (HIPEC) vs. 32.53 months (control, *p* = 0.048)	Lower peritoneal recurrence: 14% (HIPEC) vs. 43% (control)
Van Driel 2018 [15]	14.2 (HIPEC) vs. 10.7 months (control, HR 0.66, *p* = 0.003)	45.7 (HIPEC) vs. 33.9 months (control, HR 0.67, *p* = 0.02)	3-year recurrence-free: 17% (HIPEC) vs. 8% (control)
Jou 2021 [16]	11.5 (HIPEC) vs. 12.1 months (control, *p* = 0.145)	19.1 (HIPEC) vs. 30.5 months (control, *p* = 0.146)	Higher platinum-resistant recurrence: 50% (HIPEC) vs. 23% (control)
Chambers 2021 [17]	18.1 months (HIPEC: interval 15.7, recurrent 21.0)	Not reached (HIPEC)	Increased extraperitoneal recurrences (HIPEC)
Marrelli 2021 [18]	23 months (HIPEC, 5-year rate: 26%)	53 months (HIPEC, 5-year rate: 42%)	N/A
Zivanovic 2021 [19]	12.3 (HIPEC) vs. 15.7 months (controlc, HR 1.54, *p* = 0.05)	52.5 (HIPEC) vs. 59.7 months (control, HR 1.39, *p* = 0.31)	No significant differences
Kim 2022 [20]	N/A	N/A	N/A
Antonio 2022 [21]	18 (HIPEC) vs. 12 months (control, *p* = 0.038)	52 (HIPEC) vs. 45 months (control, NS)	Improved supramesocolic recurrence-free: 24.1 months (HIPEC) vs. 9.4 months (control, *p* = 0.031)
Kim-Chun 2023 [22]	HR 0.60 (HIPEC vs. control, *p* = 0.04)	HR 0.53 (HIPEC vs. control, *p* = 0.04)	Reduced platinum-resistant recurrence (HIPEC)
Lee 2023 [23]	22.9 (HIPEC) vs. 14.2 months (control, HR 0.61, *p* = 0.005)	Not reached (HIPEC) vs. 53.0 months (control, HR 0.31, *p* = 0.002)	Lower peritoneal recurrence: 32.8% (HIPEC) vs. 64.1% (control, *p* = 0.001)
Classe 2024 [24]	10.2 (HIPEC) vs. 9.5 months (control, HR 0.79, *p* < 0.05)	54.3 (HIPEC) vs. 45.8 months (control, HR 0.73, *p* = 0.024)	Reduced peritoneal progression (HIPEC)
Campos 2024 [25]	23 (HIPEC) vs. 19 months (control, *p* = 0.22)	48 (HIPEC) vs. 46 months (control, *p* = 0.579)	3-year recurrence-free survival: 47.5% (HIPEC) vs. 21.3% (control)
Ghirardi 2024 [26]	24.0 months (HIPEC)	N/A	N/A
Wang 2024 [27]	N/A	N/A	Higher CRS3 rate: 20.5% (HIPEC) vs. 4.8% (control, *p* < 0.05)
Fagotti 2024 [28]	25 (HIPEC) vs. 23 months (control)	N/A	N/A

**Table 5:** Certainty of evidence—summary of findings table.

Certainty Assessment							Certainty
No. of Studies	Study Design	Risk of Bias	Inconsistency	Indirectness	Imprecision	Other Considerations	
Progression-Free Survival							
7	Randomized trials	Serious ^a^	Serious ^b^	Not serious	Serious ^c^	None	⨁◯◯◯ Very low
Progression-Free Survival							
6	Observational studies	Very serious ^d^	Serious ^e^	Serious ^f^	Very serious ^g^	Publication bias strongly suspected strong association ^h^	⨁◯◯◯ Very low
Overall Survival							
7	Randomised trials	Serious ^i^	Not serious	Not serious	Serious ^j^	Publication bias strongly suspected ^k^	⨁◯◯◯ Very low
Overall Survival							
6	Observational studies	Very serious ^l^	Serious ^m^	Serious ^n^	Very serious ^o^	Publication bias strongly suspected strong association ^p^	⨁◯◯◯ Very low
Recurrence Patterns							
6	Randomised trials	Serious ^q^	Very serious ^r^	Serious ^s^	Serious ^t^	Publication bias strongly suspected ^u^	⨁◯◯◯ Very low
Recurrence Patterns							
6	Observational studies	Very serious ^v^	Serious ^w^	Serious ^x^	Very serious ^y^	Publication bias strongly suspected strong association ^z^	⨁◯◯◯ Very low

^a^ RCTs had some concerns regarding the randomization and deviations from intervention. ^b^ Some RCTs reported a PFS benefit while others showed no significant difference. ^c^ Sample sizes were adequate, but the variation in PFS estimates led to moderate imprecision. ^d^ Observational studies had high a risk due to confounding and selection bias. ^e^ Observational studies showed various recurrence-free survivals, which led to inconsistency. ^f^ Some observational studies had heterogeneous populations, which reduced the direct applicability. ^g^ Observational studies lacked consistency in the reported event rates and follow-up durations. ^h.^ Some observational studies may have had selective reporting favoring HIPEC. ^i^ Some RCTs had deviations from the intervention, but the overall methodology was robust. ^j^ Some RCTs had small OS sample sizes, which led to moderate imprecision. ^k^ Some RCTs did not publish long-term OS outcomes, which led to possible bias. ^l.^ Observational studies had high confounding risks, which reduced the certainty. ^m.^ Some observational studies showed an OS benefit, but with large variation in the effect sizes. ^n.^ Some studies included subpopulations that may not fully reflect real-world HIPEC use. ^o^ Wide confidence intervals in OS estimates from observational studies increased the imprecision. ^p^ Potential selective publication of positive OS outcomes in non-randomized settings. ^q^ Significant bias in the RCTs due to the inconsistent measurement of recurrence. ^r^ Recurrence patterns varied significantly across the RCTs due to different follow-up durations. ^s^ Some RCTs measured the recurrence indirectly through secondary endpoints. ^t^ Many RCTs had a limited recurrence follow-up, which increased the uncertainty. ^u^ Possible bias due to missing data on the long-term recurrence in some RCTs. ^v^ High risk of bias due to the confounding and non-random treatment assignment. ^w^ Some studies showed a HIPEC benefit while others did not, which led to a moderate inconsistency. ^x^ Observational studies had non-standardized recurrence definitions, which increased the indirectness. ^y^ Observational studies lacked uniform recurrence measurement, which led to a high imprecision. ^z^ Observational studies often lacked full reporting of the recurrence rates, which led to a moderate bias.

## Data Availability

No new data were created in this study. Data sharing is not applicable to this article.

## Abbreviations

The following abbreviations are used in this manuscript:

HIPEC	Hyperthermic intraperitoneal chemotherapy
CRS	Cytoreductive surgery
EOC	Epithelial ovarian cancer
PFS	Progression-free survival
OS	Overall survival
CC	Completeness of cytoreduction
MeSH	Medical Subject Headings
RoB	Risk of Bias
ROBINS	Risk Of Bias in Non-Randomized Studies
HGSOC	High-grade serous ovarian cancer
HRQoL	Health-related quality of life
RCT	Randomized controlled trials
HRD	Homologous recombination deficiency
